# Public health interventions for epidemics: implications for multiple infection waves

**DOI:** 10.1186/1471-2458-11-S1-S2

**Published:** 2011-02-25

**Authors:** Lindsay Wessel, Yi Hua, Jianhong Wu, Seyed M Moghadas

**Affiliations:** 1 Institute for Biodiagnostics, National Research Council Canada, Winnipeg, Manitoba, R3B 1Y6, Canada; 2 Centre for Disease Modelling, York Institute of Health Research, York University, Toronto, Ontario, M3J 1P3, Canada; 3 Department of Mathematics and Statistics, University of Winnipeg, Winnipeg, Manitoba, R3B 2E9, Canada

## Abstract

**Background:**

Epidemics with multiple infection waves have been documented for some human diseases, most notably during past influenza pandemics. While pathogen evolution, co-infection, and behavioural changes have been proposed as possible mechanisms for the occurrence of subsequent outbreaks, the effect of public health interventions remains undetermined.

**Methods:**

We develop mean-field and stochastic epidemiological models for disease transmission, and perform simulations to show how control measures, such as drug treatment and isolation of ill individuals, can influence the epidemic profile and generate sequences of infection waves with different characteristics.

**Results:**

We demonstrate the impact of parameters representing the effectiveness and adverse consequences of intervention measures, such as treatment and emergence of drug resistance, on the spread of a pathogen in the population. If pathogen resistant strains evolve under drug pressure, multiple outbreaks are possible with variability in their characteristics, magnitude, and timing. In this context, the level of drug use and isolation capacity play an important role in the occurrence of subsequent outbreaks. Our simulations for influenza infection as a case study indicate that the intensive use of these interventions during the early stages of the epidemic could delay the spread of disease, but it may also result in later infection waves with possibly larger magnitudes.

**Conclusions:**

The findings highlight the importance of intervention parameters in the process of public health decision-making, and in evaluating control measures when facing substantial uncertainty regarding the epidemiological characteristics of an emerging infectious pathogen. Critical factors that influence population health including evolutionary responses of the pathogen under the pressure of different intervention measures during an epidemic should be considered for the design of effective strategies that address short-term targets compatible with long-term disease outcomes.

## Background

Epidemics of infectious diseases have been observed throughout history, with substantial variability in their dynamical patterns. The 1918 influenza pandemic is a notorious case documented as the most devastating epidemic with over 50 million deaths and multiple outbreaks in many geographic areas worldwide [1,2]. Distinct pandemic infection waves were recorded with an 8 to 15 week interval; the latter were more severe than the first and were associated with the majority of deaths [2,3]. Although several factors may be involved, such as the effect of seasonal changes, demographics, and evolution of the virus, the true mechanism by which subsequent waves occur is not fully understood. Nor is it clearly understood how different control measures and strategies for deployment of limited health resources may interfere with disease dynamics and the occurrence of later infection waves.

Recent epidemiological and modelling studies have attempted to provide explanatory theories for the mechanisms of multiple outbreaks of an infectious pathogen capable of establishing an epidemic [2,4-10]. Spontaneous behavioural changes (e.g., a change in the number of contacts due to modified behaviour of susceptible individuals) have been shown to affect the course of infection events and produce subsequent outbreaks in an epidemic episode [9]. This has been further investigated through modelling “concerned awareness” of individuals that may result in contagion dynamics of fear and disease [6], and the implementation of public health control measures (e.g., social distancing) that may interfere with the individuals’ contact patterns during the epidemic [5]. Co-infection has also been suggested as a possible explanation for multiple infection outbreaks as a result of increased transmissibility in co-infected individuals and non-synchronicity in the time course of the two co-circulating infections [8]. Other possible mechanisms include transient post-infection immunity and evolutionary changes that may occur in the characteristics of the infectious pathogens [2,4,10].

In this study, we consider the occurrence of multiple infection waves of a pathogen from a public health perspective, and develop mathematical models to investigate how intervention measures may affect the transmission dynamics in a population. Specifically, we are interested in exploring the impact of changes in policy-relevant parameters on the patterns of disease spread during the course of an epidemic. These parameters may reflect the effectiveness of intervention strategies (e.g., treatment or isolation of infected cases) in reducing disease transmission, or their epidemiological consequences (e.g., emergence of drug resistance), and may therefore play an important role in determining the outcome of disease control activities. The significance of this work thus relates to the process of public health decision-making, in particular when confronting the emergence of a novel infectious disease with substantial uncertainty regarding the epidemiological characteristics of the invading pathogen.

For the purpose of this investigation, we develop both mean-field and stochastic epidemiological models that describe the transmission dynamics of a disease in the population, and incorporate treatment and isolation of infected cases as control measures. We parameterize these models to simulate the spread of influenza as a case study, and determine the impact of control parameters on disease dynamics. We illustrate the occurrence of multiple infection waves associated with different treatment levels and the development of drug resistance in the population under the scenario of limited capacity for treatment and isolation of infectious individuals. We compare the results obtained by simulating the mean-field model with those observed in the stochastic model, and discuss our findings in the context of epidemiology and public health.

## Methods

### The model structure

To formulate the models for describing disease epidemic, we assume that the population is initially entirely susceptible to the infectious pathogen. It is assumed that the infection can be treated with drugs, but the pathogen may develop resistance during the course of treatment with potential for transmission. Since resistance emergence may impose fitness cost on pathogen replication and transmission [11], we assume that the drug-resistant pathogen is less transmissible than the drug-sensitive pathogen. Treatment is assumed to reduce transmissibility of the drug-sensitive infection, but remains ineffective against drug-resistant infection. We also assume that the recovery from infection confers immunity to re-infection with either drug-sensitive or resistant pathogens. Considering epidemics with relatively short time-courses, we ignore the effect of recruitment, natural death, and other demographic variables of the population.

**Figure 1:**
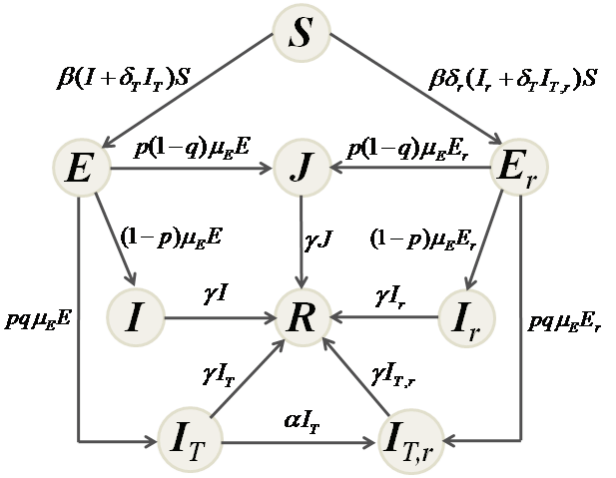
**The model.** Model diagram for the movements of individuals between population compartments.

With the assumption of homogeneous mixing, we divide the population into classes of susceptibles ( *S* ); individuals exposed (not yet infectious) to sensitive ( *E* ) and resistant ( *E_r_* ) infections; untreated individuals infected with sensitive ( *I* ) and resistant ( *I_r_* ) infections; treated individuals infected with sensitive ( *I_T_* ) and resistant ( *I_T_*_,_*_r_* ) infections; isolated individuals infected with either sensitive or resistant infection ( *J* ); and recovered individuals ( *R* ). Figure 1 shows the movements of individuals between these classes during the course of an epidemic. With parameters described in Table 1, the dynamics of the mean-field model can be mathematically expressed by the following system of differential equations:(1)

**Table 1:** Parameter values.

Parameter	Description	Value	Source
*β*	Baseline transmission rate of the sensitive infection	variable	
1/*µ_E_*	Expose period (duration between exposure and start of infectiousness)	1.25 days	[4,15-18]
1/*γ*	Duration of infectiousness	4 days	[4,15,16,18,19]
*α*	Rate of developing drug resistance following start of treatment	10^-4^ days^-1^	[16,18]
*δ_T_*	Relative transmissibility of the sensitive infection following treatment	0.4	[15,16,18,20]
*δ_r_*	Relative transmissibility of the resistant infection	0.8	[15,16,18]
*p*	Fraction of infectious individuals diagnosed for treatment or isolation	variable	
*q*	Fraction of treated infectious individuals without isolation	variable	
*T_c_*	Capacity for treatment and isolation of infectious individuals	variable	
*R*_0_	Basic reproduction number of the sensitive infection	variable	[3,19,20]

Baseline values of the parameters used for simulations of the models with sources from published literature. For a given value of *R*_0_, the baseline transmission rate *β* can be calculated using the expression *R*_0_ = *βS*_0_/*γ*.

Details of the model in its stochastic form are provided in the Appendix.

### Reproduction numbers

A key parameter in disease epidemiology is the basic reproduction number of the invading pathogen, commonly denoted by *R*_0_, which is the average number of new infections generated by a single infected case introduced into an entirely susceptible (non-immune) population [12]. The quantity *R*_0_ can be used to estimate the growth rate of an epidemic (during the initial phase) and the total number of infections (final size of the epidemic) [13]. When public health interventions are implemented, the reproduction number of disease is affected by parameters that determine the effectiveness of control measures; and we therefore introduce the control reproduction number ( *R_c_* ) to evaluate the impact of such parameters on transmissibility of the pathogen and epidemic dynamics. Applying a previously established method [12,14], for model (1), we obtain , where  and  are respectively the reproduction numbers of the sensitive and resistant infections, expressed by(2)

where *S*_0_ is the size of the susceptible population at the onset of the outbreak. In the absence of treatment and isolation, *R_c_* reduces to the basic reproduction number of the sensitive pathogen, given by *R*_0_ = *βS*_0_*/γ*. Using the expression for  in (2), one can easily calculate the critical value p^*^ at which , and therefore the spread of the sensitive infection can be contained for *p* >p^*^ . Rewriting  in terms of *R*_0_ , the value p^*^ is given by

However, the spread of disease caused by the sensitive pathogen cannot be controlled if *R*_0_ exceeds the threshold R^*^ = (*γ* + *α*)/*δ_T_ qγ*, which results in p^*^ > 1. Since 0 ≤ *q* ≤ 1, for parameter values used in simulations (Table 1), disease control becomes infeasible if *R*_0_ > 2.5. Similarly, there is a critical value  at which , and the spread of the resistant pathogen is contained if . Letting , the value of  can be expressed as

which highlights the importance of isolation for controlling the spread of resistant infection.

## Simulations and results

To simulate the models, we considered influenza infection as a case study, for which emergence and spread of drug-resistance during an outbreak can result from treatment of infected individuals. We assumed that the epidemic is triggered by a drug-sensitive influenza virus, and investigated the role of several key model parameters in changing the epidemic patterns and generating multiple waves of infection. These parameters include the fractions of infected individuals identified for treatment or isolation, and the basic reproduction number of disease which varies within the estimated range published in the literature (Table 1). Since public health resources may be limited during an epidemic, we also defined a parameter (*T_c_* ) as the capacity for treatment of infected individuals including those who are isolated (i.e., the percentage of the total population that can be treated). To illustrate various scenarios, we initially seeded a susceptible population of size *S*_0_ = 10,000 with *E*_0_ = 10 individuals exposed to the sensitive virus, and assumed that treatment can result in the emergence of resistance with the relative transmissibility *δ_r_* = 0.8 during the outbreak. Other parameter values are given in Table 1.

**Figure 2:**
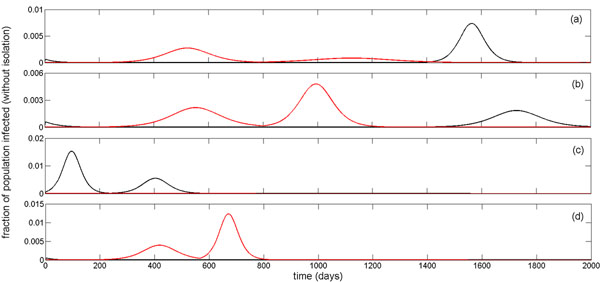
**Multiple infection waves during an epidemic episode.** Simulations for the time-courses of epidemic using parameter values given in Table 1 with: (a) *R*_0_ = 1.6; *p* = 0.68; *q* = 0.8; *T_c_* = 12% (three infection waves); (b) *R*_0_ =1.7; *p* = 0.66; *q* = 0.71; *T_c_* = 11% (three infection waves); (c) *R*_0_ = 1.9; *p* = 0.47; *q* = 0.7; *T_c_* = 18% (two infection waves); and (d) *R*_0_ = 2; *p* = 0.8; *q* = 0.64; *T_c_* = 18% (two infection waves). Black and red curves correspond respectively to the total untreated and treated sensitive ( *I* + *I_T_* ) and resistance ( *I_r_* + *I_T_*_,_*_r_*) infections without isolation.

The mean-field model was simulated for a number of scenarios to show the occurrence of multiple infection waves during an epidemic episode (Figure 2). These simulations indicate that variation in the transmissibility of the pathogen (determined by *R*_0_ ), as well as parameters that govern the effectiveness of control measures can significantly impact the epidemic profile, leading to sequences of infection waves with different magnitudes and time-courses. To explore the causes of these multiple outbreaks, we plotted time-courses of treated and untreated sensitive (black curves) and resistant infections (red curves), corresponding to epidemic profiles in Figures 2a-2d. As illustrated in Figures 2a-2b, a large scale use of treatment (combined with isolation) suppresses the spread of the sensitive infection quickly, but leads to the emergence and spread of resistance that causes the first wave of infection. Due to the limited capacity of treatment and isolation (run-out scenario), a second wave of infection follows as a result of wide-spread resistance (red curves), which declines once a sizable portion of the susceptible population is infected and the level of susceptibility reduces below a threshold that is sufficient to block the transmission of the resistant pathogen with reduced fitness. However, this level of susceptibility may still be above the threshold required for disease containment, and therefore the sensitive pathogen can cause the third wave of infection (black curves).

As the reproduction number of the sensitive infection increases (Figures 2c-2d), higher treatment levels are required for the resistant infection to prevail and cause a significant outbreak [18]. For a reduced level of treatment and a higher transmissibility of the sensitive virus, corresponding to the epidemic profile in Figure 2c, we observed two infection waves, both of which are caused by the spread of the sensitive virus, with generation of very few cases of resistant infection. In this scenario, run-out occurs before epidemic is contained, and a second infection wave takes place. Similar dynamics can occur with two subsequent waves of resistant infections for a significantly higher treatment level (Figure 2d). However, the second wave that occurs after the treatment capacity is fully dispensed (run-out scenario) leads to a major reduction in susceptibility of the population; thereby ending the epidemic. These simulations indicate that multiple infection waves could occur due to limited resources for treatment/isolation of infected cases, the ways that such resources are deployed during the outbreak, the evolutionary responses of the pathogen to control measures (e.g., emergence of drug resistance), or a combination thereof. We performed further experiments with small changes in these parameters, and observed significant influences on the epidemic dynamics that can be associated with the elimination or creation of an infection wave. It is worth noting that the above scenarios can take place even for sufficient drug stockpiles for which run-out does not occur, if a policy for adaptation (e.g., reduction) of treatment at the population level is implemented due to wide-spread drug-resistance [15].

For comparison purposes, we simulated the stochastic version of the model using a Markov Chain Monte Carlo method and observed sequences of infection waves for different sets of parameter values (see Appendix). Consistent with previous observations [4], the stochastic model displays a later peak time of infection waves (with lower magnitudes) than the homogeneous mean-field model. This depends not only on the treatment level, but also on other parameters involved in the spread of sensitive and resistant infections, such as the reduction in the potentially infectious contacts and the fitness of resistance. Furthermore, stochastic effects can play a significant role in determining disease dynamics even during the outbreak well past the initial establishment phase of the epidemic. This is illustrated in Figure 4c of the Appendix that the epidemic dies out after the first outbreak in the stochastic model; whereas a second wave of infection takes place in the mean-field model with a larger magnitude compared to the first outbreak.

**Figure 3:**
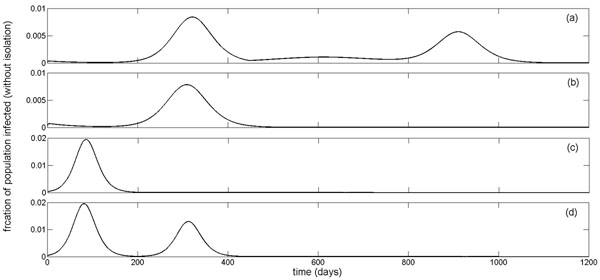
**The effect of changes in the initial number of infections on epidemic profiles.** Simulations for the time-courses of epidemic (total number of infections without isolation: *I* + *I_T_* + *I_r_* + *I_T_*_,_*_r_*) using parameter values given in Table 1. Other parameters are: (a,b) *R*_0_ =1.9; *p* = 0.72; *q* = 0.71; *T_c_* = 22.2% and initial infected cases of (a) *E*_0_ = 6 (three infection waves) and (b) *E*_0_ = 12 (single infection wave); (c,d) *R*_0_ = 2.5; *p* = 0.55; *q* = 0.41; *T_c_* = 26% and initial infected cases of (c) *E*_0_ = 8 (single infection wave) and (d) *E*_0_ = 10 (two infection waves).

In addition to parameters pertaining to the nature of disease and effectiveness of interventions, the number of infected cases at the onset of an epidemic can greatly influence the dynamics of disease. Our simulations (Figure 3) indicate that small changes in the initial number of infections may result in different epidemic profiles exhibiting more than one infection wave. This suggests that the true dynamics of an emerging disease (with unknown initial number of infections) may not be predicted with certainty, even when reliable estimates of other pathogen-related and intervention parameters are available.

## Discussion

Stellar advances in the prevention and management of infectious diseases have been achieved since the great influenza pandemic of 1918. Yet, emerging pathogens often inflict incalculable devastation to humanity. The global mobilization with rapid international transportation between populations makes the impact of such diseases even more dramatic with potential socioeconomic upheaval. This was recognized in 2003 with the appearance of severe acute respiratory syndrome (SARS) as the first major infectious disease threat of the 21^st^ century [21], and was recently experienced with the worldwide spread of a swine-origin influenza A virus H1N1, that led the World Health Organization to declare this virus as the cause of an influenza pandemic on June 11, 2009 [22]. Public health responses to the emergence of new diseases often involve difficult decisions on optimal use of health resources over very short timelines. Such decisions are further confounded by substantial uncertainties regarding the epidemiological characteristics of the novel infectious pathogen, the effectiveness of public health intervention strategies, and the evolutionary responses of the pathogen under the pressure of control measures [23]. From a population health perspective, it is therefore imperative to look beyond short-term targets and account for long-term disease outcomes in strategy development and implementation. This is particularly important for preventing multiple infection outbreaks that may result from imprudent use of resources or unintended adverse consequences of disease containment strategies.

Given the historical evidence for the occurrence of multiple infection waves [2,3,7], several modelling studies have attempted to provide explanatory theories for these events in a single epidemic course [2,5,6,8-10]. In this study, we developed mean-field and stochastic models to investigate possible causes of sequential outbreaks from a public health perspective. Our results show that epidemic dynamics can be substantially affected by factors that influence policy design and implementation (e.g., treatment level or isolation of infected individuals), and parameters that determine the effectiveness and consequences of control measures (e.g., reduction in infectiousness due to treatment or emergence of drug-resistance). Furthermore, the initial number of infections can influence disease outcomes. While mean-field and stochastic models may exhibit similar epidemic behaviour, we also observed differences in their predictions in terms of the speed with which disease spreads through the population (with further delay in the peak time of outbreaks in the stochastic model); the magnitudes of infection outbreaks; and more importantly, the occurrence of infection waves (see Appendix). The latter is particularly influenced by stochastic effects, in addition to the structure of contact patterns and heterogeneity in population interactions [4]. Previous work [4,24] provides a solid foundation for extension of this study through the development of network dynamical models of disease transmission in which heterogeneous contacts between individuals are accounted for.

In this study, we simplified the models and included compartments corresponding to some possible stages of a disease; yet we understand that different pathogens may cause infections with different clinical manifestations and infectiousness periods. For example, influenza is known to have a short latent period of less than 2 days before becoming infectious [17], followed by a pre-symptomatic infection during which disease can be transmitted without showing clinical symptoms; however, the latent period of SARS is estimated to be longer and may be comparable to the duration of a complete course of influenza infection [17]. It is also well-documented that influenza can be transmitted in asymptomatic form without developing clinical symptoms [25]; while evidence for asymptomatic transmission of SARS is rather scant. These discrepancies in infection stages of human diseases, combined with the ability of the pathogens to overcome the pressures that are applied to limit their replication and spread, can profoundly impact not only the feasibility and effectiveness of control measures, but also the dynamics of disease over the course of an epidemic. Our study highlights these considerations for further investigation, while demonstrating possible mechanisms for the occurrence of multiple infection waves in a single epidemic. Future research in this direction should address some limitations of the present study, including a systematic exploration of parameter space to characterize which intervention parameter regimes are more likely to give rise to sequences of infection outbreaks, and to determine the sensitivity of model outputs (epidemic dynamics) on parameter changes.

Although models considered here are simulated for influenza infection as a case study, understanding the interplay between intervention parameters, evolutionary responses of the pathogens, and epidemic dynamics remains a critical objective of public health for many diseases [26], including HIV, tuberculosis, malaria, and several bacterial infections. Such diseases often share common features, including the emergence and prevalence of drug resistant pathogens under the pressure of drug treatment. The initial rise of resistance is generally associated with fitness costs that make the resistant pathogen less capable of competing with the sensitive pathogen (as the dominant competitor) in a given host population [11]. However, evolutionary mechanisms (e.g., compensatory mutations [27]) may improve the fitness of resistant pathogens, and therefore intervention measures may result in further selection of resistance, as has been documented for the global spread of seasonal influenza drug resistance that appears to be associated with fitness enhancement processes [28]. This suggests that future modelling efforts should integrate factors that govern pathogen-host interactions with the mechanisms of disease epidemiology to guide public health in devising novel and effective means of infection control.

## Appendix: Stochastic model

With the same population compartments as defined in the mean-field model described in the main text, we develop a stochastic model for disease transmission dynamics to investigate the epidemic patterns with random effects. We consider time *t* as a continuous variable, and define the following random vector for *t* ∈ [0, ∞)

with  that represents changes that occur to the random vector at Δ*t* units of time. We define the transition probability as(3)

where

The function Θ(·) describes the status of an individual in a subpopulation (i.e., Θ(·) = –1: an individual leaves the subpopulation; Θ(·) = 0: no changes occur to the individuals' status in the subpopulation; Θ(·) = 1: an individual enters the subpopulation). We assume that Δ*t* is sufficiently small, so that at most one change of status can occur during the time interval Δ*t*, which can be viewed as a Markov chain process. The resulting stochastic model can be described as a continuous time Markov model, with the transition probabilities given in Table 2.

**Table 2:** Possible transitions between model compartments that can occur in Δ*t* units of time

event	transition during Δ*t*	transition rate
infection of a susceptible	*S*(*t*) → *S*(*t*) – 1	*β*(*I* + *δ_T_I_T_* + *δ_r_*(*I_r_* + *I_T,r_*))*S*(Δ*t*) + *o*(Δ*t*)
increase in exposure (sensitive infection)	*E*(*t*) → *E*(*t*) + 1	*β*(*I* + *δ_T_I_T_*)*S*(Δ*t*) + *o*(Δ*t*)
decrease in exposure (sensitive infection)	*E*(*t*) → *E*(*t*) – 1	*µ_E_E*(Δ*t*) + *o*(Δ*t*)
increase in exposure (resistant infection)	*E_r_*(*t*) → *E_r_*(*t*) + 1	*δ_r_β*(*I_r_* + *I_T,r_* )*S*(Δ*t*) + *o*(Δ*t*)
decrease in exposure (resistant infection)	*E_r_*(*t*) → *E_r_*(*t*) – 1	*µ_E_E_r_*(Δ*t*) + *o*(Δ*t*)
increase in untreated sensitive infection	*I*(*t*) → *I*(*t*) + 1	(*I* – *p*)*µ_E_E*(Δ*t*) + *o*(Δ*t*)
recovery from treated infection	*I*(*t*) → *I*(*t*) – 1	*γI*(Δ*t*) + *o*(Δ*t*)
increase in untreated resistant infection	*I_r_*(*t*) → *I_r_*(*t*) + 1	(*I* – *p*)*µ_E_E_r_*(Δ*t*) + *o*(Δ*t*)
recovery from untreated resistant infection	*I*_r_(*t*) → *I_r_*(*t*) – 1	*γI_r_*(Δ*t*) + *o*(Δ*t*)
increase in treated sensitive infection	*I_T_*(*t*) → *I_T_*(*t*) + 1	*pqµ_E_E*(Δ*t*) + *o*(Δ*t*)
recovery from treated sensitive infection	*I_T_*(*t*) → *I_T_*(*t*) – 1	(*α* + *γ*)*I_T_*(Δ*t*) + *o*(Δ*t*)
increase in treated resistant infection	*I_T,r_*(*t*) → *I_T,r_*(*t*) + 1	*pqµ_E_E*(Δ*t*) + *αI*Δ*t* + *o*(Δ*t*)
recovery from treated resistant infection	*I_T,r_*(*t*) → *I_T,r_*(*t*) – 1	*γI_T,r_*(Δ*t*)* + o*(Δ*t*)
increase in isolated infection	*J*(*t*) → *J*(*t*) + 1	*p*(1 – *q*)*µ_E_*(*E* + *E_r_*)(Δ*t*) + *o*(Δ*t*)
recovery from disease in isolation	*J*(*t*) → *J*(*t*) – 1	*γJ*(Δt) + *o*(Δ*t*)

### Algorithm for stochastic simulations

For simulating the stochastic model, we used the Markov Chain Monte Carlo method, with an initial *E*(0) = 10 exposed individuals to sensitive infection in a population of *S*_0_ = 10, 000 susceptibles. A key parameter in these simulations is the step-size of the Monte Carlo method. Using a fixed step-size requires a large number of steps to guarantee that the transitions between subpopulations take place and disease transmission can occur, which is computationally very demanding in terms of both timing and resources. To reduce such a computational load, we implemented an adaptive step-size method [29] to estimate the transition time to the next event (Δ*t*) by calculating the sum of the frequencies of all possible events, given by *η* = *β*(*I* + *δ_T_I_T_*)*S*(*t*) + *δ_r_β*(*I_r_* + *I_T,r_)S*(*t*) + (1 – *p)µ_E_*(*E* + *E_r_*) + *pqµ_E_*(*E* + *E_r_) + αI_T_* + *p*(1 – *q*)*µ_E_*(*E* + *E_r_*) + *γ*(*I* + *I_r_* + *I_T,r_* + *J* + *I_T_*). Then, by choosing Δ*t* = *U*_1_/*η*, where *U*_1_ is uniform distribution in the interval [0,1], we ordered all possible events as an increasing fraction of *η* and generated another uniform deviate (*U*_2_ ∈ [0,1]) to determine the nature of the next event. For the convergence of the results, we ran these simulations for 1000 samples, and considered the average of sample realizations of the stochastic process to generate infection curves.

### Stochastic simulations

**Figure 4:**
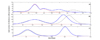
**Stochastic simulations**. Stochastic simulations for the time-courses of epidemic (including sensitive and resistant infections without isolation) using parameter values given in Table 1 of the main text with: (a) *R*_0_ = 1.9, *p* = 0.65, *q* = 0.72, *T_c_* = 19.5% (three infection waves); (b) *R_0_* = 1.9, *p* = 0.5, *q* = 0.65, *T_c_* = 15% (two infection waves); and (c) *R*_0_ = 1.9, *p* = 0.5, *q* = 0.66, *T_c_* = 16% (one infection wave). Black and red curves correspond respectively to the sensitive (untreated and treated: *I + I_τ_)* and resistant (untreated and treated: *I_r_* + *I_T_*_,_*_r_)* infections. Blue curves illustrate the corresponding scenarios for the total number of infections *(I* + *I_T_* + *I_r_* + *I_T_*_,_*_r_*) during epidemic simulated in the mean-field model. In all simulations, initial number of infected cases is *E*_0_ = 10

We ran stochastic simulations with parameter values given in Table 1 to illustrate the possibility of multiple infection waves for different scenarios with variation in the basic reproduction number, fractions of treated and isolated ill individuals, and the capacity for treatment and isolation. Figure 4a shows that, since the transmission of the sensitive infection is largely blocked by a high treatment level, resistance emerges and causes the first infection wave of the outbreak. The second wave of resistant infections follows after the capacity of treatment and isolation (*T_c_*) is exhausted, and declines when susceptibility of the population falls below a certain threshold that is sufficient to end the resistant outbreak (red curve). However, due to higher fitness of the sensitive infection, a third wave of outbreak occurs which results in depletion of the susceptible population to levels sufficient for ending the epidemic (black curve). We observed similar behaviour in the mean-field model, as illustrated by the blue curve in Figure 4a. When treatment level is reduced by a significant margin, generated resistant infection is out-competed by the sensitive infection which has a higher fitness advantage (Figure 4b), and only outbreaks of the sensitive infection occur; the second wave takes place after the capacity of treatment is fully dispensed (black curve). While, the mean-field model also produces similar results (blue curve), we observed differences in the behaviour of the stochastic model. A small reduction in the fraction of isolated individuals leads to the elimination of the second wave in the stochastic model, while mean-field model still produces a second wave with even a larger magnitude than the first wave of the outbreak (Figure 4c). This suggests that not only are stochastic effects important during the early stages of disease outset, but they also can play a critical role in shaping the epidemic well beyond the establishment phase of the disease.

## Authors’ contributions

Developed mean-field model and performed simulations: LW, SM. Developed stochastic model and performed simulations: YH, SM. Designed the study and wrote the paper: JW, SM. All the authors have read the final version of the paper and approved it.

## Competing interests

The authors declare that they have no competing interests.
